# Alaska ecosystem carbon fluxes estimated from MODIS satellite data inputs from 2000 to 2010

**DOI:** 10.1186/1750-0680-8-12

**Published:** 2013-11-22

**Authors:** Christopher Potter, Steven Klooster, Vanessa Genovese

**Affiliations:** 1 NASA Ames Research Center, Mail Stop 232-21, Moffett Field, CA 94035, USA; 2 California State University Monterey Bay, Seaside, CA, USA

**Keywords:** Net carbon flux, MODIS EVI, Ecosystems, Alaska

## Abstract

**Background:**

Trends in Alaska ecosystem carbon fluxes were predicted from inputs of monthly MODerate resolution Imaging Spectroradiometer (MODIS) vegetation index time-series combined with the NASA-CASA (Carnegie Ames Stanford Approach) carbon cycle simulation model over the past decade. CASA simulates monthly net ecosystem production (NEP) as the difference in carbon fluxes between net primary production (NPP) and soil microbial respiration (Rh).

**Results:**

Model results showed that NEP on a unit area basis was estimated to be highest (> +10 g C m^-2^ yr^-1^) on average over the period 2000 to 2010 within the Major Land Resource Areas (MRLAs) of the Interior Brooks Range Mountains, the Arctic Foothills, and the Western Brooks Range Mountains. The lowest (as negative land C source fluxes) mean NEP fluxes were predicted for the MLRAs of the Cook Inlet Lowlands, the Ahklun Mountains, and Bristol Bay-Northern Alaska Peninsula Lowlands. High levels of interannual variation in NEP were predicted for most MLRAs of Alaska.

**Conclusions:**

The relatively warm and wet years of 2004 and 2007 resulted in the highest positive NEP flux totals across MLRAs in the northern and western coastal locations in the state (i.e., the Brooks Range Mountains and Arctic Foothills). The relatively cold and dry years of 2001 and 2006 were predicted with the lowest (negative) NEP flux totals for these MLRAs, and likewise across the Ahklun Mountains and the Yukon-Kuskokwim Highlands.

## Background

Climate is changing worldwide, but Alaska is warming at a rate almost twice the global average [1]. Changes already observed in Alaskan landscapes include rapidly eroding shorelines, melting ground ice (permafrost), wetland drying, ice wedge degradation, increased shrub growth at high latitudes, and conifer forest decline [2-4]. Sustainable ecological function and threatened wildlife species are at risk throughout Alaska [5,6].

Satellite remote sensing has been shown to be an accurate method to monitor large-scale change of vegetation cover, especially following disturbance [7-10]. There have been numerous previous studies of satellite greenness index patterns in Alaska and arctic North America. For example, Jia et al. [11] analyzed 21 years (1981–2001) of AVHRR-NDVI (Advanced Very High Resolution Radiometer - Normalized Difference Vegetation Index) data for three bio-climatic subzones in northern Alaska and confirmed a long-term trend of increase in vegetation greenness for the Alaskan tundra. This study reported a 17% increase in peak vegetation greenness across the region (corresponding to simultaneous increases in air temperatures), and field sampling throughout the region revealed that NDVI explained over 82% of total above-ground plant biomass. Goetz et al. [12,13] analyzed the seasonal and inter-annual variations of post-fire forest cover by using AVHRR-NDVI time-series across boreal North America and reported vegetation compositional changes consistent with early successional plant species and susceptibility to drought. Beck and Goetz [14] reported that increases in tundra productivity from satellite observations for the North Slope of Alaska do not appear restricted to areas of high shrub cover, and that enhanced productivity was found across mixed vegetation types that include graminoids [15].

Other mechanisms of change in tundra and boreal ecosystems have also been studied with remote sensing. In areas on the North Slope of Alaska where topography strongly controlled the flow and redistribution of surface water, NDVI change was found to be strongly related to the variability in the depth of the active (thawed) soil layers of tundra [16]. Kim et al. [17] further examined changing soil freeze–thaw signal from satellite microwave remote sensing and vegetation greenness patterns for the 9-year (2000–2008) vegetation record from satellites over North America, and reported that the relationship between the non-frozen period (June–August) and mean summer greenness index anomalies was generally positive for tundra and boreal forest areas of Canada.

Previous simulation modeling studies of carbon storage for Alaska estimated that terrestrial ecosystems have been a net sink (from the atmosphere) of between 5 and 12 Tg C (1 Tg = 10^12^ grams) yr^-1^ for the state land area in the 1980s, and between 0 and 10 Tg C yr^-1^ during the 1990s [18,19]. Using a combination of field measurements and modeling approaches, O’Donnell et al. [20] estimated typical net accumulation rates of 23 g C m^-2^ yr^-1^ and 57 g C m^-2^ yr^-1^ in permafrost landforms and peat bog ecosystems, respectively, over the last 100 years in interior Alaska. Nonetheless, when wetland methane (CH_4_) emissions were also considered (and expressed in CO_2_ equivalents) in the regional carbon budget, Zhuang et al. [19] estimated that Alaska’s terrestrial ecosystems functioned as large net source of carbon greenhouse gases to the atmosphere during the period of 1980 to 1996.

In the present study, we utilized the Enhanced Vegetation Index (EVI) image data from NASA’s MODerate resolution Imaging Spectroradiometer (MODIS) satellite sensor as inputs to the CASA (Carnegie Ames Stanford Approach) ecosystem carbon model for the state of Alaska from 2000 to 2010. The main research questions addressed in this study were:

•*How has climate variability altered patterns of ecosystem carbon storage in Alaska, and what were the primary mechanisms driving these changes?*

•*Which ecosystems and landscape components in Alaska were most vulnerable to abrupt loss of ecosystem carbon over the past decade, and which were the most resistant to abrupt loss of ecosystem carbon?*

## Results

### CASA validation with Alaska tower flux measurements

Flux estimates from eddy-correlation analysis were obtained from tower flux sites in Alaska that could meet certain criteria for CASA model comparisons [21]. First, at least three complete years of site flux measurements were required to evaluate model predictions of interannual variations in CASA NEP fluxes. Second, winter season NEP fluxes were required from a site to evaluate model predictions of soil CO_2_ emissions on a year-round basis. Third, tower sites were required to be representative of the predominant vegetation class setting in the MODIS land cover data used as input to the CASA model [22].

For sites meeting all of these criteria, data sets were obtained from the central data repository located at the Carbon Dioxide Information Analysis Center (CDIAC; http://public.ornl.gov/ameriflux/dataproducts.shtml). Level 4 AmeriFlux records contained gap-filled and *μ*star filtered records, complete with calculated gross productivity and total ecosystem respiration terms on varying time intervals including hourly, daily, weekly, and monthly with flags for the quality of the original and gap-filled data.

CASA monthly NEP predictions from the MOD13C2 EVI data values closest to the tower location were compared to AmeriFlux eddy-correlation monthly estimates of the corresponding NEP fluxes. We note that the monthly MODIS EVI values in practically every grid cell of the global CASA model will be influenced by periodic land cover disturbances and (some naturally occurring) areas of sparse vegetation cover, including development, roads, water bodies. It was expected, therefore, that CASA model NEP flux predictions would be systematically lower than tower measurements of these carbon fluxes, since tower footprints tend to be far less affected by historical wildfire and other disturbances (such as logging and forest thinning), compared for instance to the surrounding MODIS grid cell area in which they are located.

**Figure 1:**
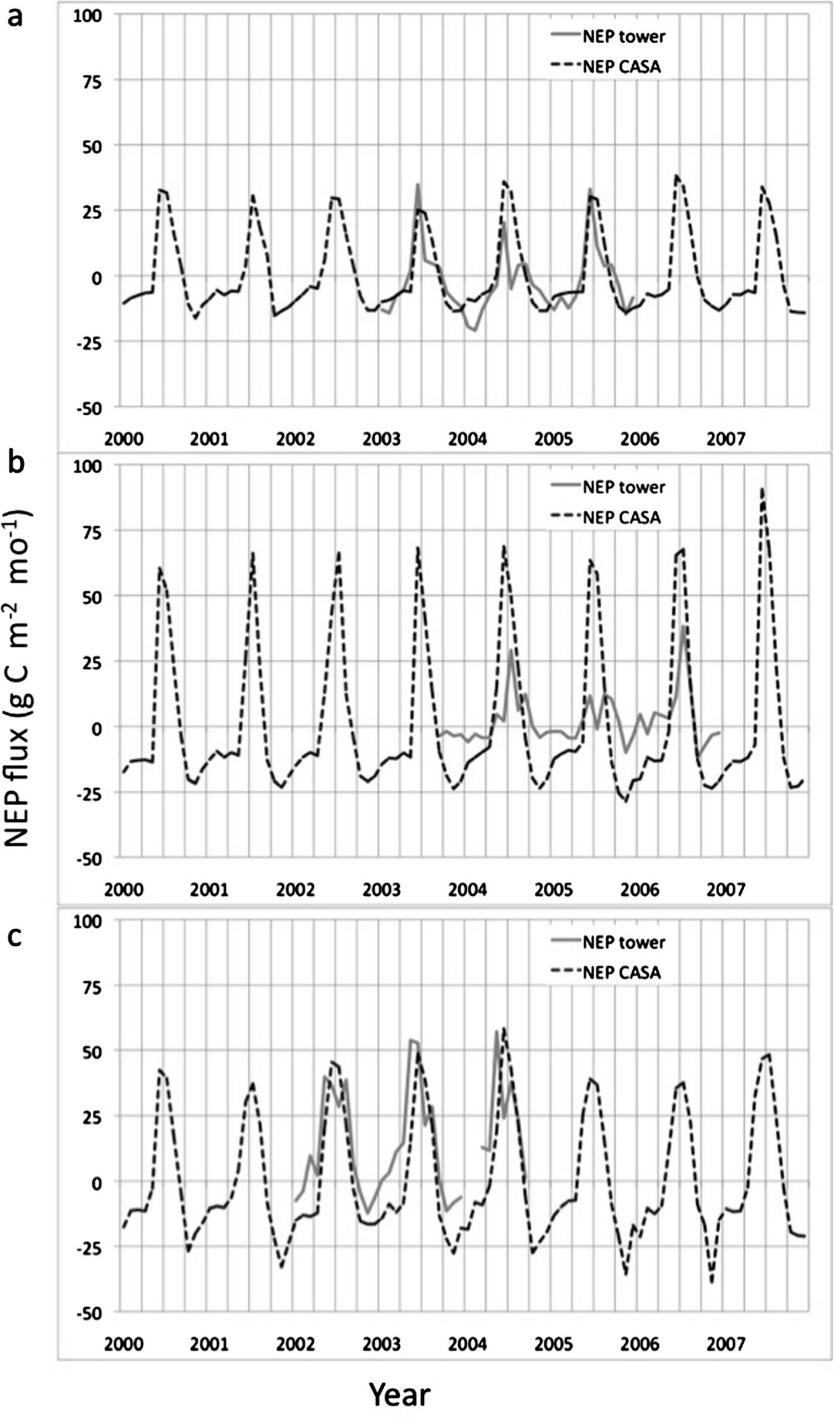
**CASA monthly NEP predictions in comparison to compared to AmeriFlux eddy-correlation monthly estimates. (a)** Atqasuk wetland, **(b)** Ivotuk shrubland, **(c)** Delta Junction forest.

**Figure 2:**
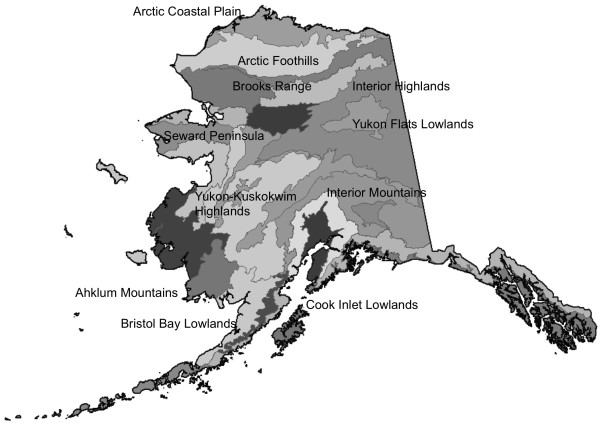
Major Land Resource Areas (MLRAs) of Alaska, with labels for the areas cited in this study.

A total of three AmeriFlux tower sites in Alaska (listed in Table 1), together reporting 107 monthly NEP flux measurements, were found to meet the criteria cited above for comparison to CASA model NEP predictions. Results showed that the CASA model predictions closely followed seasonal timing of tower measurements at each site (Figure 1). The linear regression correlation coefficient (*R*^*2*^) between CASA model NEP predictions and tower fluxes NEP was estimated at between *R*^*2*^ = 0.44 and 0.60 (significant at *p* < 0.05) for the three sites. We note that the Level 4 AmeriFlux data reported for monthly NEP at the Ivotuk open tundra site in the Arctic Foothills (MLRA; [23], Figure 2) were not consistent in seasonal sign (as ecosystem source or sink of carbon flux) from year to year, which rendered those measured fluxes far less reliable for model comparisons than the other two AmeriFlux tower sites listed in Table 1. Previous publications have reported that these *R*^*2*^ results are typical of model-tower flux comparisons, in large part because eddy flux measurements carry large uncertainties into the comparisons [24,25].

**Table 1:** AmeriFlux tower sites in Alaska selected for comparison to CASA monthly NEP flux predictions

**Sitename**	**Latitude**	**Longitude**	**Elevation (m)**	**Vegetation cover**	***R***^***2 ***^**for CASA NEP**
Atqasuk	70.4696	−157.4089	15	Permanent wetlands	0.60
Ivotuk	68.4870	−155.7480	568	Open shrublands	0.44
Delta Junction 1920 control	63.8881	−145.7394	518	Evergreen needleleaf forests	0.65

### Alaska ecosystem carbon fluxes from 2000 to 2010

**Figure 3:**
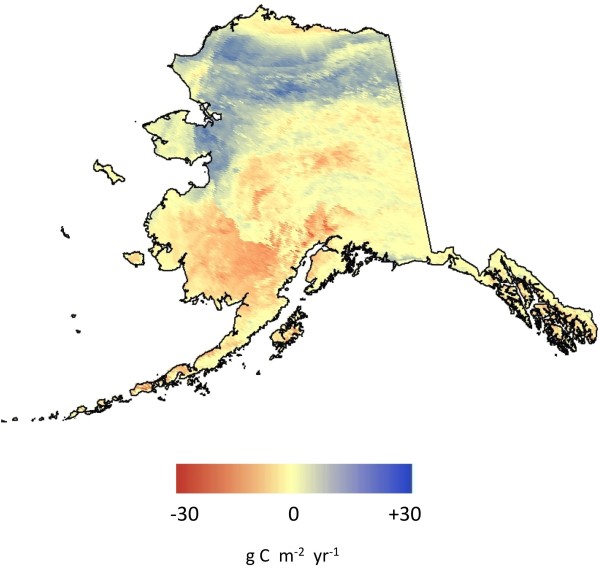
CASA model prediction of mean annual NEP flux in Alaska over the years 2000 to 2010.

CASA annual NEP on a unit area basis was estimated to be highest (> +10 g C m^-2^ yr^-1^) on average over the period 2000 to 2010 within the Major Land Resource Areas (MRLAs) of the Interior Brooks Range Mountains, the Arctic Foothills, and the Western Brooks Range Mountains (Table 2 and Figure 3). The lowest (negative) mean NEP fluxes from 2000 to 2010 were predicted for the MLRAs of the Cook Inlet Lowlands, the Ahklun Mountains, and Bristol Bay-Northern Alaska Peninsula Lowlands, all in excess of −4 g C m^-2^ yr^-1^. The highest variability from year-to-year in predicted NEP flux was estimated within the Seward Peninsula and Yukon-Kuskokwim Highlands MLRAs.

**Table 2:** CASA mean annual NEP flux predictions over the period 2000 to 2010 by the MLRAs in Alaska, sorted by highest (positive: land sink) to lowest (negative: land source) total mean carbon flux

**Major Land Resource Area**	**Area**	**Mean**	**Standard deviation**	**Total (Mean × area)**
	**km**^**2**^	**g C m**^**-2**^ **yr**^**-1**^	**g C m**^**-2**^ **yr**^**-1**^	**g C yr**^**-1**^
Arctic Foothills	108,544	12.09	4.93	1.3E + 12
Interior Brooks range mountains	48,768	13.67	4.39	6.7E + 11
Western Brooks range mountains	59,648	10.59	5.50	6.3E + 11
Interior Alaska highlands	178,624	1.89	5.22	3.4E + 11
Northern Brooks range mountains	41,216	7.94	5.12	3.3E + 11
Nulato Hills-Southern Seward Peninsula highlands	46,400	6.76	7.67	3.1E + 11
Seward Peninsula highlands	34,368	8.26	7.20	2.8E + 11
Northern Seward Peninsula-Selawik lowlands	20,736	9.00	5.68	1.9E + 11
Upper Kobuk and Koyukuk hills and valleys	34,240	2.86	4.02	9.8E + 10
Arctic Coastal plain	58,496	1.27	4.08	7.4E + 10
Southern Alaska coastal mountains	67,392	0.33	1.97	2.2E + 10
Copper River Basin	11,840	0.36	1.69	4.3E + 09
Northern Bering Sea Islands	9,216	−0.72	2.53	−6.6E + 09
Yukon Flats lowlands	32,704	−0.49	2.61	−1.6E + 10
Southern Alaska Peninsula mountains	15,680	−1.26	2.59	−2.0E + 10
Interior Alaska mountains	114,496	−0.30	2.78	−3.5E + 10
Northern Alaska Peninsula mountains	14,400	−2.47	2.59	−3.6E + 10
Kodiak Archipelago	12,352	−3.10	3.68	−3.8E + 10
Aleutian Islands-Western Alaska Peninsula	25,024	−2.96	4.71	−7.4E + 10
Cook Inlet mountains	51,008	−1.98	3.80	−1.0E + 11
Alexander Archipelago-Gulf of Alaska Coast	66,944	−1.60	3.92	−1.1E + 11
Interior Alaska lowlands	93,696	−1.21	3.99	−1.1E + 11
Cook Inlet lowlands	27,328	−6.19	4.33	−1.7E + 11
Ahklun mountains	37,760	−5.27	2.59	−2.0E + 11
Yukon-Kuskokwin coastal plain	76,864	−2.68	2.78	−2.1E + 11
Bristol Bay-Northern Alaska Peninsula lowlands	50,176	−4.51	3.15	−2.3E + 11
Yukon-Kuskokwim highlands	155,072	−3.16	5.95	−4.9E + 11
State total	1,492,992			2.4E + 12

**Figure 4:**
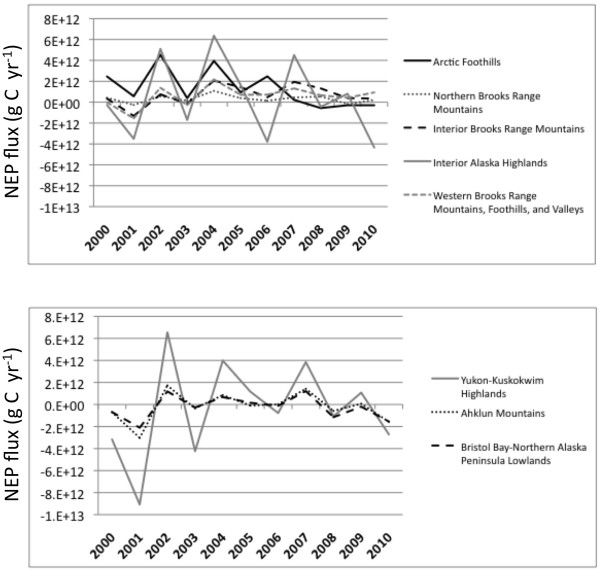
Yearly (2000 to 2010) variability in CASA model predicted NEP for selected MLRAs in Alaska.

The CASA model predicted three years (2002, 2004, and 2007) during which the highest positive (land C sink) NEP total fluxes totaled to between +2 to +6 Tg C yr^-1^ across individual MLRA regions (Figure 4). Three other years (2001, 2003, and 2006) were predicted with the lowest negative (land C source) NEP total fluxes at between −2 to −9 Tg C yr^-1^ across individual MLRA regions (Figure 4). Not withstanding this high level of interannual variation, CASA mean annual NEP flux over the period 2000 to 2010 for all MLRAs in Alaska was estimated at +2.4 Tg C yr^-1^ (Table 2).

**Figure 5:**
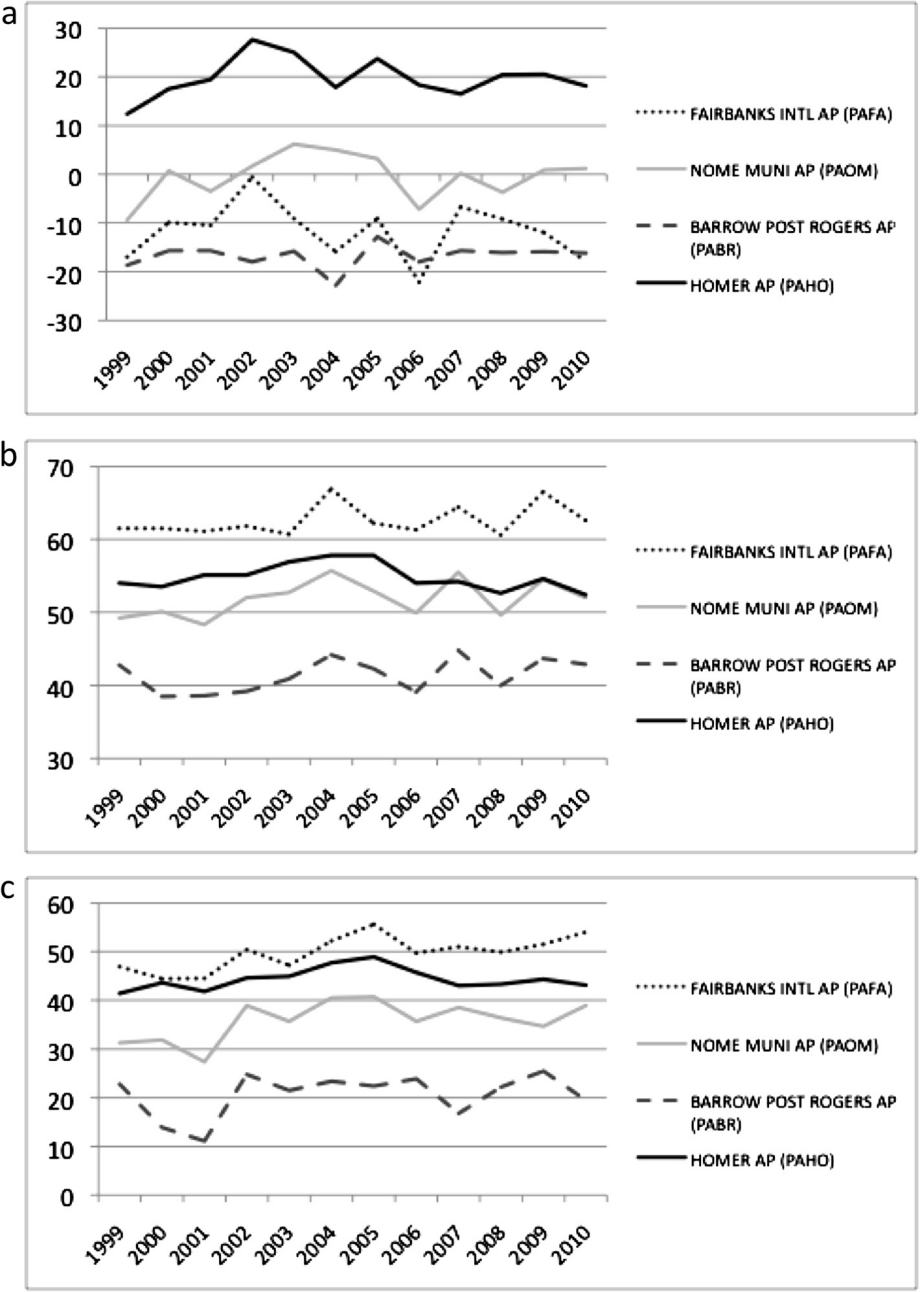
Surface temperature variations (2000 to 2010, in degrees F) from selected Alaska weather stations, (a) minimum, (b) maximum, (c) May of each year.

**Figure 6:**
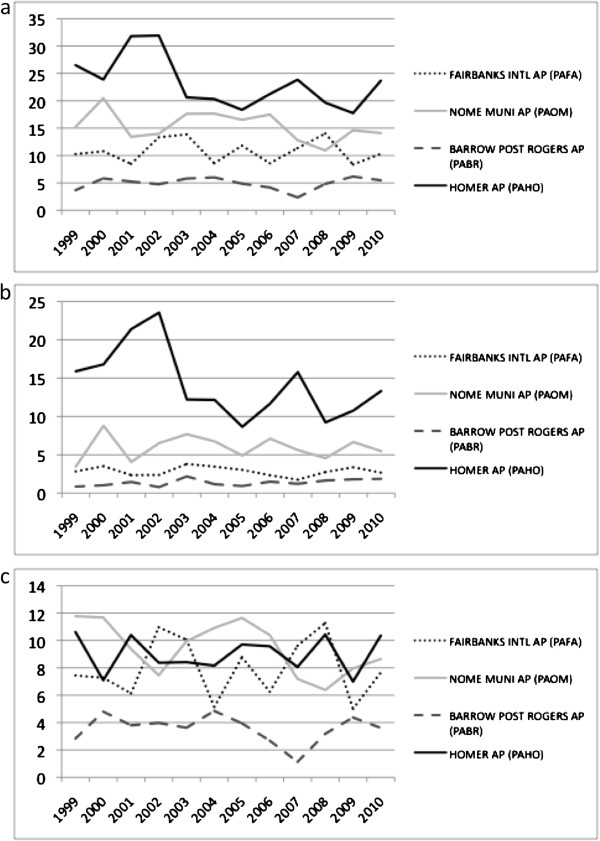
Total precipitation variations (2000 to 2010, in inches) from selected Alaska weather stations, (a) annual, (b) winter, (c) summer.

To help account for the interannual variation in predicted NEP fluxes, four station locations were selected from among the NOAA National Weather Service Aviation Weather Center archive (http://aviationweather.gov) records to represent the climatic and geographic extents of the state, namely the station records from Homer, Nome, Fairbanks, and Barrow Alaska. The years 2002, 2004–2005, and 2007 showed the warmest surface temperature departures over the past decade at all four stations (Figure 5). Temperature departures during 2001 and 2006 were recorded as the coldest over the past decade at all four stations. Precipitation records showed 2001 to be the wettest year over the past decade at the Homer station, whereas 2004–2005 was the wettest period at the Nome and Barrow stations (Figure 6). The 2006 to 2007 period was among the driest at the Barrow station. Precipitation at the Fairbanks station varied from wettest in 2002 and 2003 to driest in 2004 and 2009.

## Discussion

The CASA model results and MODIS EVI time-series data used in this study provided consistent large-scale metrics of vegetation growth and carbon flux trends across the arctic region. We hypothesize from the modeling results presented here that temperature warming-induced inter-annual variability of evapotranspiration (ET) at local and regional scales has altered the rates of ecosystem carbon exchange in Alaska over recent years. In the CASA model, warming-enhanced ET flux during the growing season would increase NEP fluxes during wet years, and would decrease NEP fluxes during dry years ([21,26]). In further support of this hypothesis, evidence of shrub expansion over the last half century has been documented through repeat photography [27]. Beck and Goetz [14] also postulated that the proportion of North America tundra areas increasing in productivity has steadily grown since 1982, reaching 32% of non-barren areas in 2008. This regional greening trend appeared to be unrelated to shrub density, indicating that primary productivity is increasing across a range of functional vegetation types.

Recent studies have indicated that wildfires in Alaska can have a large impact on ecosystem carbon stocks, a disturbance flux which we have not addressed in this CASA modeling study. For instance, Yuan et al. [28] reported that soil C stocks would have increased by 158 Tg C if the Yukon River Basin (YRB) had not undergone warming and changes in fire regime since the 1960s. Tan et al. [29] estimated that YRB wildfires resulted in the total emission of 81 ± 14 Tg C yr^-1^ in 2004 alone, of which 73% and 27% could be attributed to the consumption of the ground layer and aboveground biomass, respectively. This fire emission was equivalent to 61% of the annual biomass C photosynthesized over the YRB study area, assuming the average ecosystem net primary production (NPP) was 266 g C m^-2^ in 2004 [30].

Previous simulation modeling studies of carbon storage for Alaska estimated that terrestrial ecosystems have been a net sink (from the atmosphere) of between +5 and +12 Tg C in the 1980s, and between 0 and +10 Tg C yr^-1^ during the 1990s [18,19]. The CASA model results since 2000 presented in this study indicated that there has been a high level of interannual variability within those previously estimated ranges, fluctuating around an average NEP flux total of +2.4 Tg C yr^-1^.

In answer to the question of how climate variability has affected patterns of ecosystem carbon storage in Alaska, we may conclude from the CASA model results that the relatively warm and wet years of 2004 and 2007 resulted in the highest positive NEP flux totals across MLRAs in the northern and western coastal locations in the state (i.e., the Brooks Range Mountains and Arctic Foothills). The relatively cold and dry years of 2001 and 2006 were predicted with the lowest (negative) NEP flux totals for these MLRAs, and likewise across the Ahklun Mountains and the Yukon-Kuskokwim Highlands.

In answer to the question of which ecosystems or landscape components in Alaska were most vulnerable to abrupt loss of ecosystem carbon over the past decade, it was evident from the CASA model results presented here that the Interior Highlands and the Yukon-Kuskokwim Highlands have experienced the greatest annual declines and interannual variations in NEP total flux over the past decade. As for which ecosystems were the most resistant to abrupt loss of ecosystem carbon, we can hypothesize from the CASA model results that the Arctic Foothills ecosystems have not released as much CO_2_ to the atmosphere during warm/dry periods as have other MLRAs in Alaska.

It should be noted that Wang et al. [31] reported on sensor degradation having had an impact on trend detection in North America boreal and tundra zone NDVI with Collection 5 data from MODIS. The main impacts of gradual blue band (Band 3, 470 nm) degradation on simulated surface reflectance was most pronounced at near-nadir view angles, leading to a small decline (0.001–0.004 yr^−1^; 5% overall between 2002 and 2010) in NDVI under a range of simulated aerosol conditions and high-latitude surface types. Even if this same sensor degradation problem affected MODIS EVI trends over the period of our analysis from 2000 to 2010 (which has not been reported in a publication), the apparent rate of greening in ecosystems of Alaska was evidently not negated by such small, progressive changes in MODIS data quality [32].

In closing, the CASA modeling methodology developed for mapping and characterization of ecosystem carbon fluxes can be readily extended over the upcoming decade of Collection 6 MODIS EVI data. The next set of CASA runs for Alaska will incorporate new statewide mapping of wetland types developed by Whitcomb et al. [33] to generate 1-km resolution flux estimates of methane (CH_4_) emissions. These simulations will follow the CASA wetland NEP modeling methods published by Potter et al. [34] for the continental United States coverage. We will thereby be well-positioned to answer the research question of how land surface hydrology patterns (specifically related to wetlands) interact with vegetation, topography, and permafrost to influence both CO_2_ and CH_4_ emissions in northern latitude ecosystems.

## Methods

Monthly NPP of vegetation from the CASA model was predicted using the relationship between greenness reflectance properties and the fraction of absorption of photosynthetically active radiation (fPAR), assuming that net conversion efficiencies of PAR to plant carbon can be approximated for different ecosystems or are nearly constant across all ecosystems [35-37]. For this study, we used MODIS collection 5 of the Enhanced Vegetation Index (EVI; [38]) as model inputs for PAR interception, aggregated for regional assessments to an 8-km spatial resolution.

As documented in Potter [39-41], monthly production of plant biomass is estimated as a product of time-varying surface solar irradiance, Sr, and EVI (for fPAR) from the MODIS sensor, plus a constant light utilization efficiency term (emax) that is modified by time-varying stress scalar terms for temperature (T) and moisture (W) effects (Equation 1).

(1) NPP=SrEVIemaxTW

The CASA emax term was set uniformly at 0.55 g C MJ^-1^ PAR, an approach that derives from calibration of predicted annual NPP to previous field estimates [42]. This model setting has been successfully validated globally by comparing predicted annual NPP to more than 1900 field measurements of NPP [43], and against numerous Fluxnet eddy covariance tower site measurements of NPP from 2000–2007 [26]. Gridded monthly climate inputs for these CASA runs were from National Center for Environmental Prediction (NCEP) reanalysis products (version NCEP/DOE II; [44,45]).

The CASA model is designed to couple daily and seasonal patterns in soil nutrient mineralization and soil heterotropic respiration (Rh) of CO_2_ from soils. The CASA soil carbon model uses a set of compartmental difference equations [43]. First-order decay equations simulate exchanges of decomposing plant residue (metabolic and structural fractions) at the soil surface. The model also simulates surface soil organic matter (SOM) fractions that presumably vary in age and chemical composition. Turnover of active (microbial biomass and labile substrates), slow (chemically protected), and passive (physically protected) fractions of the SOM are represented. CASA includes boreal forest floor organic layers (ground moss) and soil freeze/thaw (permafrost) dynamics in its simulation of ecosystem carbon flux [46,47]. The soil pools are initialized for global CASA model runs with a 3600 monthly time step “spin-up” routine, based on average monthly climate drivers representing the late 1990s.

Monthly and annual NEP was computed as NPP minus Rh fluxes, excluding the contributions of forest fires and other localized disturbances to ecosystem CO_2_ source emissions. Global CASA NEP predictions have been validated previously against numerous temperate and tropical ecosystem measurements of NEP from eddy covariance tower sites from 2000–2007 [21]. Sensitivity and uncertainty analysis has been conducted previously for the CASA model in northern ecosystems, along with numerous other carbon models as part of the NASA-BOREAS campaign [48].

Collection 5 MODIS data sets beginning in the year 2000 were obtained from NASA’s Land Processes Distributed Active Archive Center site [49]. MODIS EVI values were aggregated to 8-km resolution from MOD13C2 (MODIS/Terra Vegetation Indices) products. MOD13C2 data are cloud-free spatial composites of the gridded 16-day 1-kilometer MOD13A2 product, and were provided monthly as a level-3 product projected on a 0.05 degree (5600-meter) geographic Climate Modeling Grid (CMG). Cloud-free global coverage at 8-km spatial resolution was achieved by replacing clouds with the historical MODIS time-series EVI record. MODIS EVI was calculated from red, blue and NIR bands as described by Huete et al. [50]. MODIS EVI was scaled from its original values to a range of 0 to 1 for CASA model inputs.

## Competing interests

The authors declare that they have no competing interests.

## Authors’ contributions

All authors have made substantial contributions to the acquisition of data, analysis and interpretation of results, drafting the manuscript, and have given final approval of the version to be published.
